# High-throughput formation and image-based analysis of basal-in mammary organoids in 384-well plates

**DOI:** 10.1038/s41598-021-03739-1

**Published:** 2022-01-10

**Authors:** Soojung Lee, Jonathan Chang, Sung-Min Kang, Eric Parigoris, Ji-Hoon Lee, Yun Suk Huh, Shuichi Takayama

**Affiliations:** 1 Wallace H. Coulter Department of Biomedical Engineering, Georgia Institute of Technology, Atlanta, GA USA; 2 The Parker H. Petit Institute of Bioengineering and Bioscience, Georgia Institute of Technology, Atlanta, GA USA; 3 Department of Green Chemical Engineering, Sangmyung University, Cheonan, Chungnam 31066 Republic of Korea; 4 Department of Biological Engineering, NanoBio High-Tech Materials Research Center, Inha University, 100 Inha-ro, Incheon, 22212 Republic of Korea

**Keywords:** High-throughput screening, Assay systems

## Abstract

This manuscript describes a new method for forming basal-in MCF10A organoids using commercial 384-well ultra-low attachment (ULA) microplates and the development of associated live-cell imaging and automated analysis protocols. The use of a commercial 384-well ULA platform makes this method more broadly accessible than previously reported hanging drop systems and enables in-incubator automated imaging. Therefore, time points can be captured on a more frequent basis to improve tracking of early organoid formation and growth. However, one major challenge of live-cell imaging in multi-well plates is the rapid accumulation of large numbers of images. In this paper, an automated MATLAB script to handle the increased image load is developed. This analysis protocol utilizes morphological image processing to identify cellular structures within each image and quantify their circularity and size. Using this script, time-lapse images of aggregating and non-aggregating culture conditions are analyzed to profile early changes in size and circularity. Moreover, this high-throughput platform is applied to widely screen concentration combinations of Matrigel and epidermal growth factor (EGF) or heparin-binding EGF-like growth factor (HB-EGF) for their impact on organoid formation. These results can serve as a practical resource, guiding future research with basal-in MCF10A organoids.

## Introduction

In the past few decades, 3D organoid research has been actively conducted and has resulted in many remarkable achievements^1–6^. Nonetheless, the reproducibility and accessibility of these 3D models is still a major challenge in the field^7–10^. We have previously demonstrated the formation of organoids in a one-drop, one-organoid format using human non-tumorigenic mammary MCF10A cells^11^. To generate MCF10A organoids, a specialized hanging drop culture plate was required. These organoids display large diameters with hollow lumens and express mammary gland-specific and progenitor markers, thus resembling characteristics of human mammary tissue. The hanging drop methodology consistently produces large, single organoids and contrasts with conventional gel scaffold culture platforms and overlay systems where MCF10A cells form small, variably sized acini^12–14^. A key technical aspect of our method that drives this difference is the use of a minimal Matrigel scaffold—named for its orders of magnitude less use of Matrigel compared to conventional methods. Furthermore, we discovered that these MCF10A organoids, generated with the minimal scaffold, possess a basal-in phenotype where the basement membrane is formed on the inner surface of the MCF10A epithelium^15^. This phenotype is the opposite of traditional organoids and provides easier analysis of cancer cell invasion^15^. While several reports of so-called inverted organoids exist^16–19^, prior systems observe much smaller structures that often have mixed polarity and form through eversion of an initially basal-out polarity organoid^20–22^. Our hanging drop method produces organoids with a stable basal-in phenotype and does not require eversion^15^.

**Figure 1:**
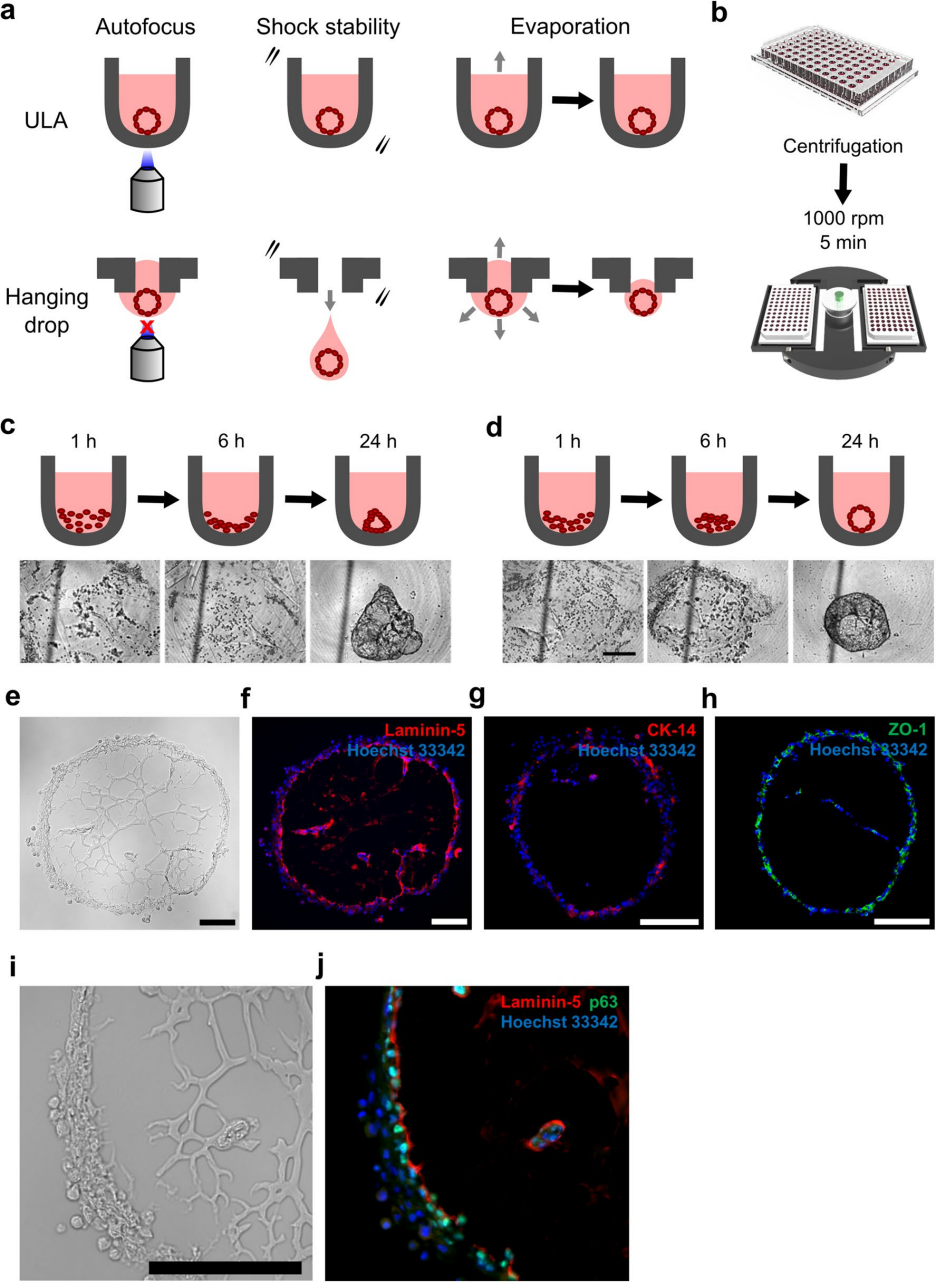
384-well ultra-low attachment (ULA) plates combined with centrifugation allow for basal-in mammary organoid formation. (**a**) The ULA plate format provides several key advantages over hanging drop: (1) compatibility with autofocusing microscopy systems, (2) increased resistance to physical disturbances, and (3) reduced evaporation. (**b**) Schematic showing 384-well ULA plate centrifugation at 1000 rpm (142 rcf) for 5 min. (**c**) Schematic and images of cell-Matrigel suspension in a ULA well without centrifugation. (**d**) Schematic and images of ULA well with centrifugation. Scale bar represents 400 μm. (**e**–**j**) Brightfield and immunofluorescence images from sections of day 16 organoids formed in the 10 ng/mL EGF and 120 µg/mL Matrigel. All scale bars represent 150 μm.

While the hanging drop platform allows for reliable formation of organoids, it suffers from a lack of accessibility and requires a high degree of technical expertise to implement (Fig. 1a). Hanging drop plates are susceptible to organoid loss due to plate disturbance, more prone to evaporation, and difficult to obtain from commercial sources. Additionally, it is challenging to autofocus on cells within hanging drops. These combined limitations have hindered our efforts to use various commercial automated imaging platforms with hanging drop plates, and instead, we resort to manual and labor-intensive imaging. This constraint has precluded our ability to analyze the early stages of organoid formation and other relatively fast (time scale on the order of hours) organoid structure changes which require frequent image capture. In an effort to improve compatibility with automated imagers, we attempted but failed to produce large MCF10A organoids in a one-well, one-organoid format using commercial 96-well ULA plates^11^. We hypothesized that these 96-well ULA plates failed due to a lack of curvature-guided cell aggregation. Therefore, we fabricated custom polydimethylsiloxane (PDMS) microwells with varying curvature to systematically test this hypothesis. Indeed, we observed successful single organoid formation when the microwells were sufficiently curved for gravity-driven cell aggregation. Building on this finding, we combined commercially available 384-well U-bottom ULA (from here on described simply as 384-well ULA) plates—which are more curved than the previously tested 96-well version—with an added centrifugation step to promote cell aggregation (Fig. 1b). This new method still utilized a minimal Matrigel scaffold and successfully produced basal-in MCF10A organoids. Furthermore, it allowed in-incubator imagers to capture time points more frequently: every 1–2 h compared to the once or twice daily possible with hanging drop plates. Together with a custom algorithm for automated measurement of organoid size and circularity, we utilize this broadly accessible 384-well ULA platform to rapidly screen combinations of Matrigel and growth factor that are most effective and reliable in producing basal-in MCF10A organoids in a one-well, one-organoid format.

## Results

### MCF10A organoid formation in curvature-controlled PDMS wells

To test the hypothesis that better cell and material aggregation will promote organoid formation, we fabricated PDMS microwells of constant diameter and varying concave curvatures. The different curvatures were generated by coating flat bottom wells with uncured tert-butanol (tBA)-PDMS mixtures. Curing of the PDMS along with evaporation of tBA resulted in the formation of a curved surface at the bottom of the well due to capillary force filling the corners. The PDMS microwells became more curved with increasing percentages of tBA in the mixture (see Supplementary Fig. S1 online). By comparing MCF10A organoid formation in these PDMS microwells, we found that the more curved microwells were able to form single, circular organoids (see Supplementary Fig. S1 online). This success of the highly curved PDMS microwells became a cornerstone for evaluating the potential of commercial 384-well ULA plates to form basal-in MCF10A organoids.

### Basal-in MCF10A organoid formation in commercial 384-well ULA plates

Understanding that higher curvature promotes successful organoid formation, we sought an appropriate commercial microwell plate. The well curvature in 384-well ULA plates approaches that of the successful PDMS microwells and is considerably more curved than the previously and unsuccessfully tested 96-well ULA plate^11^ (see Supplementary Fig. S2 online). While initial results using the commercial 384-well ULA plates were encouraging, MCF10A organoid formation was still slow and produced less circular organoids (Fig. 1c). Therefore, we added an initial centrifugation step to further promote cell aggregation and quickly form uniform rounded structures (Fig. 1d) with high yield (96%). A video showing how the initial centrifugation step promotes cell aggregation and early sphere formation is available in Supplementary Video S1 online. Next, we analyzed whether the MCF10A organoids produced displayed a basal-in phenotype. Figure 1e–j represents brightfield and immunostaining images of organoids obtained from culture in 10 ng/mL EGF and 120 µg/mL Matrigel. The organoid sections were stained with laminin-5 (red), an indicator of cell-produced basement membrane. It is noted that laminin-5 is not present in significant amounts in Matrigel^12,14,23,24^. Consistent with our previous publication with hanging drop^15^, the staining showed localization of laminin-5 to the inner surface of the MCF10A epithelium, thereby confirming the formation of basal-in mammary organoids in 384-well ULA plates (Fig. 1f). The resulting organoids consist of basal-like epithelial cells as indicated by the cytokeratin-14 (red) and p63 (green) immunostainings (Fig. 1g,j). The junction marker ZO-1 was stained with a diffuse pattern similar to MCF10A acini (Fig. 1h)^25^. Luminal epithelial cell marker, cytokeratin-8 and other basal markers (i.e. calponin-1 and MYH11) were not detected.

### Live-cell imaging and development of an automated image analysis script for organoid analysis

**Figure 2:**
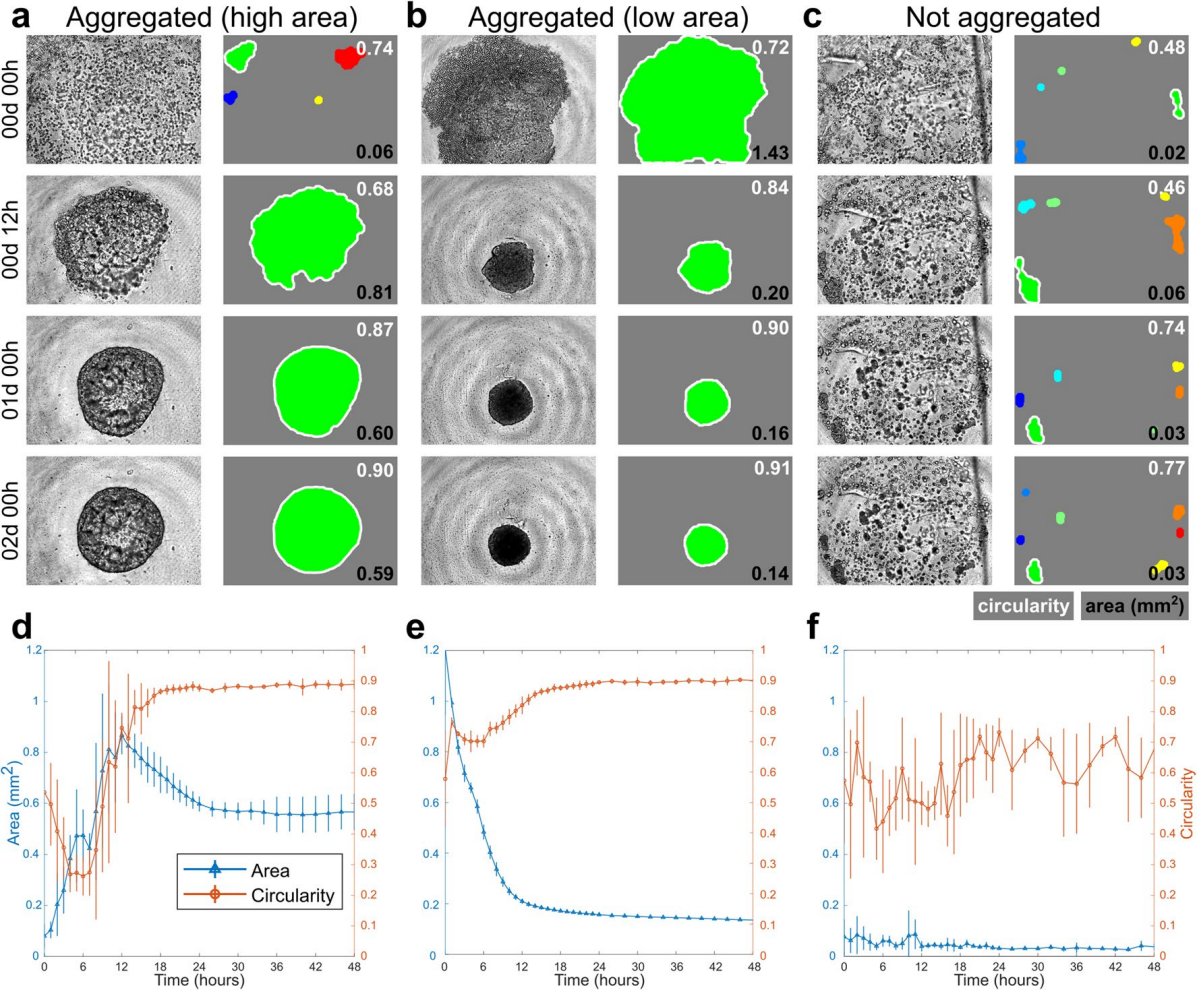
Image analysis comparing aggregated (high area), aggregated (low area), and not aggregated structures within the first 48 h. Original and processed images are shown for representative (**a**) aggregated (high area) (200 μg/mL Matrigel and 100 ng/mL HB-EGF), (**b**) aggregated (low area) (no Matrigel and 100 ng/mL HB-EGF), and (**c**) not aggregated (200 μg/mL Matrigel and 0.1 ng/mL HB-EGF) structure formation over the first 48 h. Only the green object outlined in white (the largest structure) is quantified. Area and circularity measurements for (**d**) aggregated (high area), (**e**) aggregated (low area), and (**f**) not aggregated structure formation over time are calculated averages from quadruplicate experiments (shown as mean ± sd).

Capitalizing on the ULA plate’s compatibility with in-incubator live-cell imaging, we utilized an Incucyte S3 (Sartorious) microscope system to more frequently (up to 1 scan per hour) image our forming organoids. The Incucyte S3 is capable of autofocusing on and individually imaging each well in the 384-well ULA plate, and therefore, tens of thousands of images can accumulate over the course of a single experiment. One major challenge for analysis is handling this massive amount of image data. Here, we developed an automated MATLAB script based on morphological image processing to extract the projected area (from here on described simply as area) and measure the circularity of the largest cellular structure in each image (Fig. 2a–c). Incucyte timing constraints precluded us from performing whole well scans. Thus, we were limited to using the 10× objective which reduced our field of view. The majority of cellular structures were still captured in our images, but some were only partially captured. In particular, large, off-centered organoids could extend slightly beyond the image field of view. Supplementary Fig. S3 online depicts some of these organoids which were considered as partial spheres.

### Tracking of early structural changes in aggregated and not aggregated conditions

The combination of more frequent imaging and automated analysis uniquely enables us to track structural changes with enhanced temporal resolution. Therefore, we analyzed the first 48 h—the time period of greatest structural change—for three experimental conditions. For comparison, we selected two conditions which successfully aggregated into highly circular, single structures with high and low areas (Fig. 2a,b), and one condition which did not aggregate (Fig. 2c). These conditions were 200 μg/mL Matrigel + 100 ng/mL HB-EGF, no Matrigel + 100 ng/mL HB-EGF, and 200 μg/mL Matrigel + 0.1 ng/mL HB-EGF respectively. These conditions showcase the broad range of cellular structures which can result depending on the initial concentrations of Matrigel and growth factor, and demonstrate how altering just one of these variables can drastically change the resulting cellular structure—an anecdotal observation also noted from our previous hanging drop work^11,15^.

Observing the changes in area and circularity over the first 48 h (Fig. 2d–f), we see noticeable differences in profile. For the circularity time course, both aggregated conditions show a rapid increase in circularity which plateaus to a high value around 0.9: a perfect circle would have a value of 1. The not aggregated condition displays sporadic changes in circularity over time ranging from approximately 0.4–0.7. For the aggregated (high area) condition, the area displays a peak in size during the initial 24 h of culture (Fig. 2d), which differs from the aggregated (low area) condition which rapidly decreases in area during the initial 24 h (Fig. 2e). These aggregation profile differences may delineate between hollow lumen formation and dense compaction; visual inspection of the final structures (Fig. 2a,b at t = 2 days) seems to support this hypothesis, but further experimentation is required. The not aggregated condition maintains a small size for the entire time course (Fig. 2f).

To explain these differences in area and circularity profiles, we can reference the processed images at select time points. For the aggregated (high area) condition at t = 0 h, the cell and extracellular matrix (ECM) material is widely dispersed throughout the entire well, and our image processing algorithm identifies many small discrete objects. Since only the largest contiguous object is analyzed, the starting images all result in low area values. Figure 2a at t = 12 h displays the initial aggregation for this condition. For the aggregated (low area) condition, aggregation is faster; our algorithm can detect a single contiguous object from the very first image (Fig. 2b at t = 0 h), thus explaining the high initial area. For the not aggregated condition, irregularly shaped cell clumps form throughout the experiment and never aggregate into a single mass (Fig. 2c). These clumps vary widely in their circularity, and this is shown in the higher variance values for the not aggregated condition. While it is possible for a not aggregated structure to contain a circular cell clump, this structure is not maintained consistently throughout the time course. Moreover, since aggregation often occurs during the first 24 h of culture, we were previously not able to observe these processes. In hanging drop culture, images are only taken every few days to avoid plate disturbance and to minimize the frequency of tedious manual imaging.

### High-throughput screen of Matrigel and growth factor combinations

**Figure 3:**
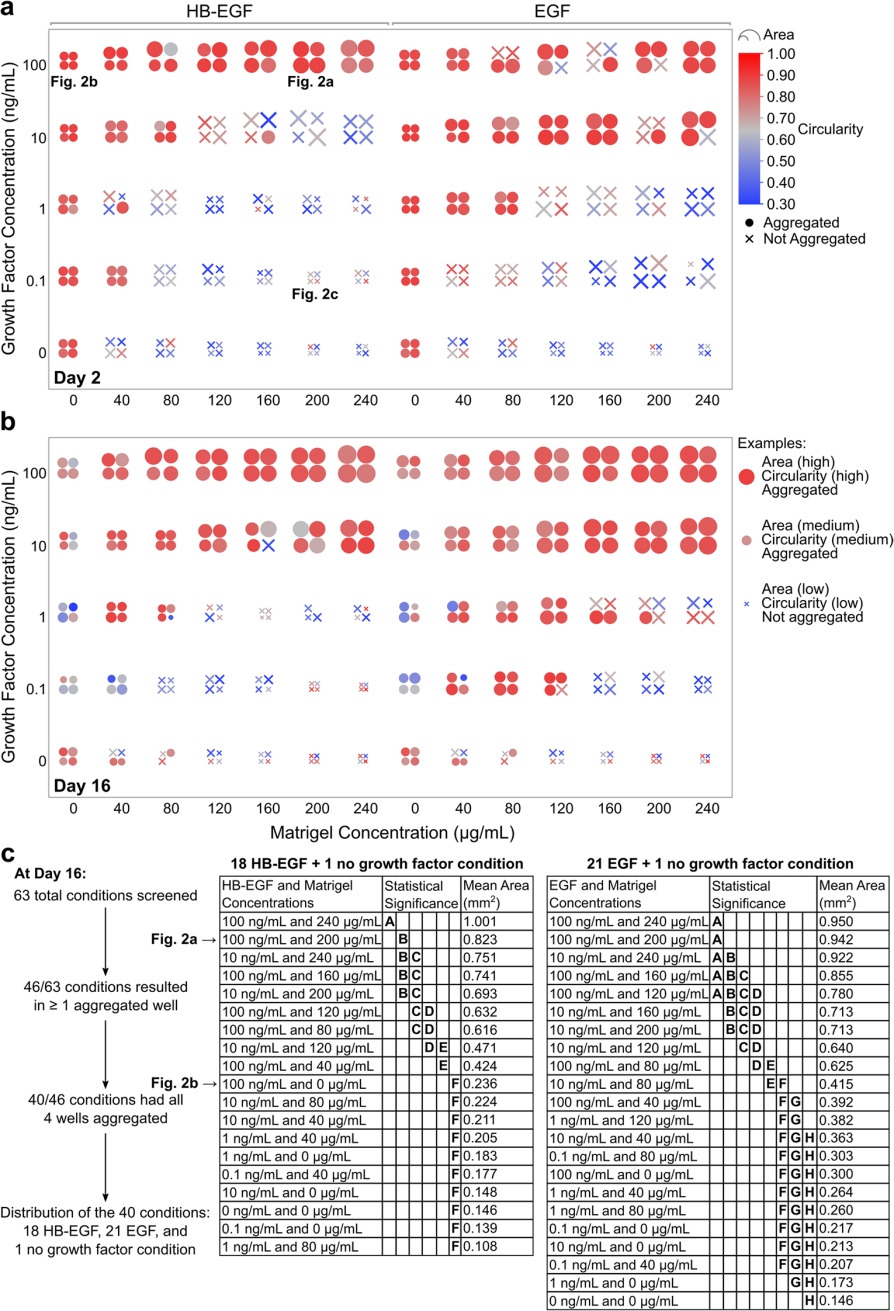
Screening the effects of Matrigel and growth factor concentration on organoid formation. Summary of organoid formation under 63 different Matrigel and growth factor combinations at (**a**) day 2 and (**b**) day 16. Projected area, circularity, and whether the structure is aggregated or not aggregated are represented by the marker size, color, and shape respectively. Each marker represents a different replicate in a quadruplicate study. Conditions are divided by growth factor type (HB-EGF: left, EGF: right), and the no growth factor conditions are duplicated on both sides for ease of visualization. (**c**) Statistical analyses of the most consistent conditions with 4 out of 4 wells aggregated at day 16 are shown. Conditions not connected by the same letter have significantly different areas (ANOVA and post-hoc Tukey test: P < 0.05). Conditions depicted in Fig. 2 are labeled.

With our new ULA-based system, a broad screen of 63 distinct combinations of Matrigel and growth factor—with concentrations ranging from 0–240 μg/mL and 0–100 ng/mL, respectively—was performed in a quadruplicate experiment (Fig. 3). We tested two of the most commonly used growth factors for MCF10A cell culture: HB-EGF and recombinant human-EGF (EGF). Images after 2 and 16 days of culture were manually labeled to denote if a fully aggregated structure had successfully formed, and area and circularity measurements were calculated using our image analysis protocol (see Supplementary Fig. S4 online). After 2 days (Fig. 3a), 34 out of the total 63 conditions (54%) had at least one replicate form an aggregated structure. Of these 34 conditions, 26 (76%) had all four wells form aggregated structures. In contrast, at day 16 (Fig. 3b), 46 out of the total 63 conditions (73%) had at least one replicate aggregated, and of these 46 conditions, 40 (87%) had all four replicates successfully aggregate. These results show that the majority of structures will fully aggregate in the first 2 days. However, some conditions—particularly those with higher Matrigel concentrations—may take longer. A comparison of conditions with a constant growth factor concentration of 10 ng/mL in Fig. 3a,b demonstrates this effect. Additionally, in general, increasing either Matrigel or growth factor concentration will increase the size of the aggregated structure; a finding which corroborates previous results^11,15^. Yet, arbitrarily increasing either one of these variables alone will not produce the largest possible organoids. Balance between Matrigel and growth factor concentrations is necessary for successful aggregation (i.e. sufficient growth factor levels must support increases in Matrigel). Observation of this effect can be seen in the 1 ng/mL growth factor conditions in Fig. 3b.

Finally, among the best performing conditions—those which all four replicates aggregated by day 16—we conducted statistical analyses to identify significant differences in structure area (Fig. 3c). Here, we have provided lists of these conditions divided by growth factor type and sorted by descending mean area to serve as a practical resource for future basal-in MCF10A organoid studies where careful selection of organoid size may be required. These results also have utility in conserving expensive reagents. For example, the 10 ng/mL EGF and 240 μg/mL Matrigel condition is able to produce similarly sized organoids (i.e. not significantly different) to the 100 ng/mL EGF and 240 μg/mL Matrigel condition while using only a tenth of the amount of growth factor. On a larger scale, this type of insight could result in substantial cost-savings in both academic and industry settings.

## Discussion

We describe the development of an alternative and technically more robust method (Fig. 1a) to form basal-in MCF10A mammary organoids using 384-well ULA plates combined with a centrifugation step (Fig. 1b). Additionally, we simultaneously present a custom MATLAB script designed specifically for handling the increased image load. Together, we believe these advancements circumvent the factors—such as technically-demanding handling, droplet evaporation, and labor-intensive imaging—which may preclude broad adoption of our hanging drop technology; the use of commercially available microwell plates, a staple in biological research, greatly improves the accessibility of our basal-in MCF10A model. Moreover, we have broadly tested and optimized this platform over 63 conditions spanning several fold concentrations of Matrigel and growth factor, and we have produced practical lists of some of the most consistent conditions for producing fully aggregated structures. However, we recognize that our image analysis protocol is limited to only measuring organoid area and circularity and that other features may be important to function. For instance, high EGF concentrations will produce organoids with partially cell-filled interiors (see Supplementary Fig. S5 online)—an observation only seen after organoid sectioning. Future adaption of our analysis methodology to include more advanced image processing techniques could help to tease out these differences.

Enabled by the augmented temporal resolution of our system, we demonstrate as a proof-of-concept the analysis of rapid structural changes during early cell and ECM material aggregation, but this tracking can be expanded to observe other events which cause structural changes on short time scales (hours). Moreover, based on PDMS microwell experiments, this system should be scalable to even higher-throughput well plates (e.g. 1536 well) and could have industry applications. These higher-throughput plates are more curved and therefore are expected to aggregate cells and extracellular materials without centrifugation (see Supplementary Fig. S2 online). Nonetheless, further optimization would be needed, and these studies may be limited by the capabilities of the microscope system.

While the present work focused on basal-in MCF10A organoid formation, the methodology presented could be expanded to work with other cell types. We believe this work will accelerate future discoveries in 3D organoid research.

## Material and methods

### 2D cell culture

MCF10A cells were purchased from the American Type Culture Collection (ATCC) and were cultured in growth media containing DMEM/F12 (Gibco #11330-032, lot 2186800) and supplemented with 5% horse serum (Gibco #16050-122, lot 2104932), 20 ng/mL HB-EGF (Peprotech #100-47, lot 0618325 I0319), 0.5 µg/mL hydrocortisone (Sigma #H0888, lot SLBT5910), 100 ng/mL Cholera toxin (Sigma #C8052, lot 019M4023V), and 10 µg/mL insulin (Sigma #I1882, lot SLCB0128)^15^. 2D cultures were maintained at 37 °C and 5% CO_2_ in T75 culture flasks, supplemented with 1% penicillin/streptomycin (Gibco #15140-163, lot 2289325), and routinely passaged at 70–80% confluence.

### 3D organoid culture

To culture MCF10A mammary organoids in 3D ULA culture, cells were seeded in 384-well U-bottom ULA plates (S-bio #MS-9384UZ, lot 90639317), centrifuged at 1000 rpm for 5 min (142 rcf, Thermo Scientific Sorvall ST16 centrifuge), and maintained in the plate using the various culture conditions (Fig. 3). Briefly, 3000 MCF10A cells were seeded in each well in a final volume of 25 μL. The cells were supplemented with 0.24% methocel A4M (Sigma #94378, lot BCBR9701V), 0–240 µg/mL Matrigel (Corning #356231, 8.5 mg/mL, lot 9301006), 10% FBS (GeminiBio #900-108, lot A52G00J), and 0–100 ng/mL growth factor, HB-EGF or EGF (Peprotech #AF-100-15, lot 1020AFC05). On day 3 of organoid culture, the media was exchanged 3 times to wash out the seeding supplements using a CyBio FeliX liquid handling machine (Analytik Jena). This culture media was supplemented with 0–100 ng/mL growth factor depending on condition and was exchanged 2 times every 2–3 days. Organoids were maintained in culture for 16 days.

### Fabrication of PDMS microwells of varying concave curvature

The master mold for a cylindrical microwell array was produced by a micro-milling machine (Minitech Machinery Corp., CNC Mini-Mill/GX). The design of an acrylic master mold was created using SOLIDWORKS, which was converted to G-code for operating the micro-milling machine. In this process, a 0.039-mm diameter square endmill was used to generate the post of cylinders.

The PDMS cylindrical microwell array was fabricated using conventional soft lithography methods^26,27^. Briefly, a mixture of 10:1 PDMS prepolymer and curing agent (Dow Corning, 184 Sylgard Silicon Elastomer) was poured over the master mold, degassed for 1 h in a vacuum chamber, and cured at 70 °C for 12 h. After curing, the PDMS replica was carefully peeled off the master mold. The diameter and height of the fabricated cylindrical microwell were 2 mm and 2.5 mm respectively.

The varying concave curvature in these PDMS microwells was generated by using capillarity-induced solvent evaporation. In detail, an uncured PDMS mixture of prepolymer and curing agent in a 10:1 ratio was dissolved in tBA (Sigma-Aldrich, 308250) with varying final percentages of tBA: 5, 10, 15, and 20%. Then, the tBA-PDMS mixture was poured onto the cylindrical microwell plate and incubated at 70 °C for 12 h. Solvent evaporation and curing produced the PDMS structures with differing curvatures depending on the tBA percentage. The resulting PDMS microwells were used for 3D organoid culture after UV sterilization.

To measure PDMS well curvature, raw micrographs were analyzed using ImageJ to quantify bottom and top well diameters and well depths. Curvature values were calculated from the measured bottom diameters. These values were compared to curvature values calculated for commercial 96-, 384-, and 1536-well plates; well dimensions obtained from Corning Inc. (catalog numbers: 4515, 4516, and 4527, respectively) were used for calculation (see Supplementary Fig. S2).

### Real-time cell imaging of morphological changes in different ULA culture conditions

We tested, in quadruplicate, a combination of 63 different culture conditions with varying Matrigel concentrations and the proliferative growth factor EGF or HB-EGF. The various conditions for real-time cell imaging in ULA culture are depicted in Supplementary Fig. S3 online. While the organoids were maintained for 16 days in ULA culture, they were imaged using an Incucyte S3 (Sartorious) in-incubator microscope system. The Incucyte S3 autofocuses on each well of the microwell plate. 10× brightfield images were acquired at 1 h intervals for the first day, every 2 h for second day, and then every 4 h for the remainder of the experiments. For early time-lapse tracking of circularity and area, images from the first 48 h were exported and analyzed in MATLAB using our custom script. For analysis of all 63 tested conditions, images from day 2 and day 16 were exported and analyzed in MATLAB. JMP was utilized to generate the plots seen in Fig. 3a,b. Additionally, one-way ANOVAs and post-hoc Tukey tests were performed in JMP using a significance level of 0.05.

### Image analysis

A custom script in MATLAB was prepared to automatically analyze each organoid image. First, threshold segmentation was used to separate each organoid from its background and to create a binarized image. Then, these images were inverted and a disc-shaped structuring element with a radius of 30 pixels was used to perform a morphological opening process followed by a closing step. This filtering process helped to remove pixels that did not belong to the organoid. The bwboundaries function in MATLAB was then used to trace the boundaries of each organoid. From the identified objects, the regionprops function was used to find the area and circularity of each organoid. The area was taken as the number of pixels contained within an organoid, and circularity was calculated from the Eq. (1) below.

1 $$Circularity=\frac{4\pi \times Area}{Perimete{r}^{2}}$$

For not aggregated culture conditions that resulted in several smaller clusters, these values were extracted from the largest object in each image.

### Sample embedding and cryosectioning

Organoids were collected from the ULA plate upon completion of the experiment on day 16, and washed with PBS. Samples were fixed in 4% paraformaldehyde for 30 min at room temperature and washed three times in PBS^15^. To aid in the visualization of the organoids, they were stained with 0.5% methylene blue (RICCS Chemical Company #4850-4, lot 2905C60) for 10 min, followed by PBS washes to remove the excess dye. A small amount of optimal cutting temperature (OCT, Tissue-Tek #62550-01, lot 190710) was added to a cryomold, and 4 organoids were added to each mold. Subsequently, the organoids were covered with additional OCT. Isopentane (Sigma #M32631, lot SHBK7564) was cooled in liquid nitrogen, and samples were flash frozen in the isopentane for less than 2 min. Cryoblocks were stored at − 80 °C, and 10 μm sections of the organoids were obtained using a CryoStar NX70 cryostat (Thermo Fisher).

### Immunofluorescence and microscopy

Mounted sections were thawed for 5 min, and a hydrophobic pen was used to outline the regions of interest. The sections were rinsed with PBS, and permeabilized with 0.2% Triton X-100 (Sigma #T8787, lot SLBV4122) for 5 min as previously described^15^. Briefly, the sections were washed 3 times with PBS, and blocked with 4% bovine serum albumin solution (BSA, Millipore Sigma #82-067) in PBS for 1 h at room temperature. Mouse monoclonal anti-laminin 5 (Abcam #ab78286, clone #P3H9-2, lot no. GR3360269-2, diluted 1:200), mouse monoclonal anti-cytokeratin-14 (Abcam #ab7800, lot no. GR3335210-1, diluted 1:1000), mouse monoclonal anti-cytokeratin-8 (Santa Cruz Biotechnology #sc-8020, diluted 1:100), rabbit polyclonal anti-Zo-1 (Invitrogen #61-7300, diluted 1:50), mouse monoclonal anti-MYH11 (Santa Cruz Biotechnology #sc-6956, diluted 1:400), and rabbit monoclonal anti-calponin-1 (Abcam #ab46794, lot no. GR3250050-5, diluted 1:200) primary antibodies were prepared in a 1% BSA solution. The 4% BSA was removed and the primary antibody solutions were added to the samples, and incubated at 4 °C overnight. The primary antibody was then removed, and samples were washed with 1% BSA in PBS solution 3 times for 5 min each. Goat anti-mouse IgG conjugated with Alexa Fluor 647 (Invitrogen #A32728, lot UK290265, diluted 1:500) and anti-rabbit (Cell signaling #4412 s, diluted 1:500) secondary antibodies were prepared in a 1% BSA solution and incubated with the samples for 2 h at room temperature. Slides were rinsed 3 times with PBS, and incubated with Hoechst 33342 (Invitrogen #H3570, 10 mg/mL, lot 2096796, diluted 1:5000) for 10 min at room temperature. The slides were rinsed 3 times with PBS, briefly dried, and mounted using ProLong Diamond Antifade mounting media (Invitrogen #P36961, lot P36961). A DMi8 inverted epifluorescence microscope (Leica) equipped with 20× and 40× air objectives was used to image the samples.

## Supplementary Information

Supplementary Information 1.

Supplementary Video S1.

## Data Availability

The datasets generated and/or analyzed during the current study for the purpose of this article are available from the corresponding author on reasonable request.

## Code availability

We have provided our MATLAB script as a Supplementary Method online.
