# Supplementary material for: High-throughput formation and image-based analysis of basal-in mammary organoids in 384-well plates
Source: Sci Rep. 2022 Jan 10;12:317. doi: 10.1038/s41598-021-03739-1 (PMC8748891; doi:10.1038/s41598-021-03739-1)
Supplement: Supplementary file 1 — Supplementary Information 1. [file 41598_2021_3739_MOESM1_ESM.docx]

High-throughput formation and image-based analysis of basal-in mammary organoids in 384-well plates

Soojung Lee^1,2,5^, Jonathan Chang^1,2,5^, Sung-Min Kang^3^, Eric Parigoris^1,2^, Ji-Hoon Lee^1,2^, Yun Suk Huh^4^, and Shuichi Takayama^1,2,^*

Supplementary Information

1. Supplementary Figure S1

MCF10A organoid generation in curvature-controlled PDMS microwells. (A) PDMS microwells with different curvatures were fabricated by creating cylindrical cavities, filling the cavity with PDMS diluted to different extents, then allowing the solvent to evaporate (represented by arrows) as the PDMS coats the walls. (B) Micrographs of PDMS microwell cross-sections and different curvatures obtained with different tBA dilutions. Dotted white arrows point to regions where sharp corners become more rounded. Higher curvature microwells form better organoid structure. All scale bars represent 500 μm.


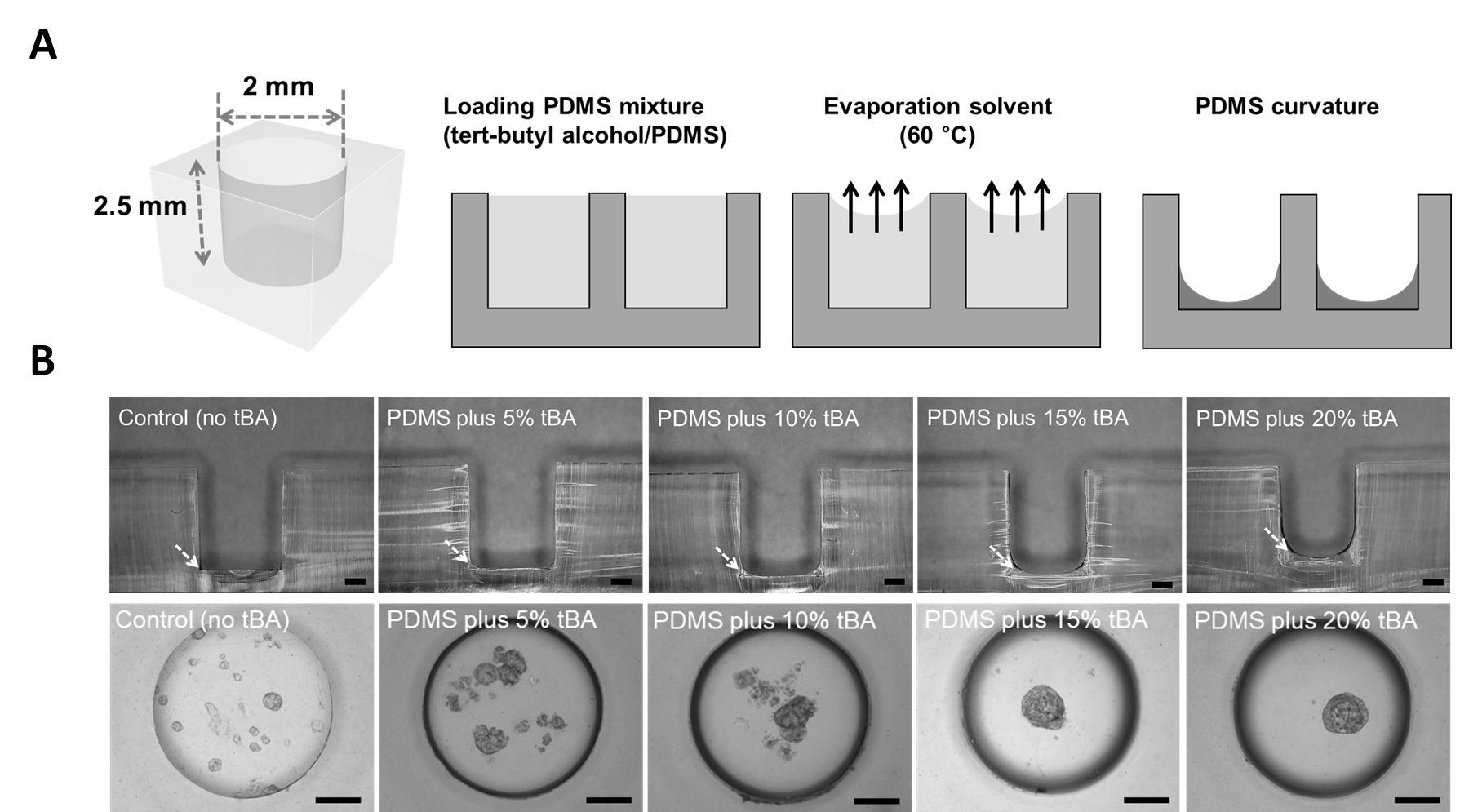


2. Supplementary Figure S2


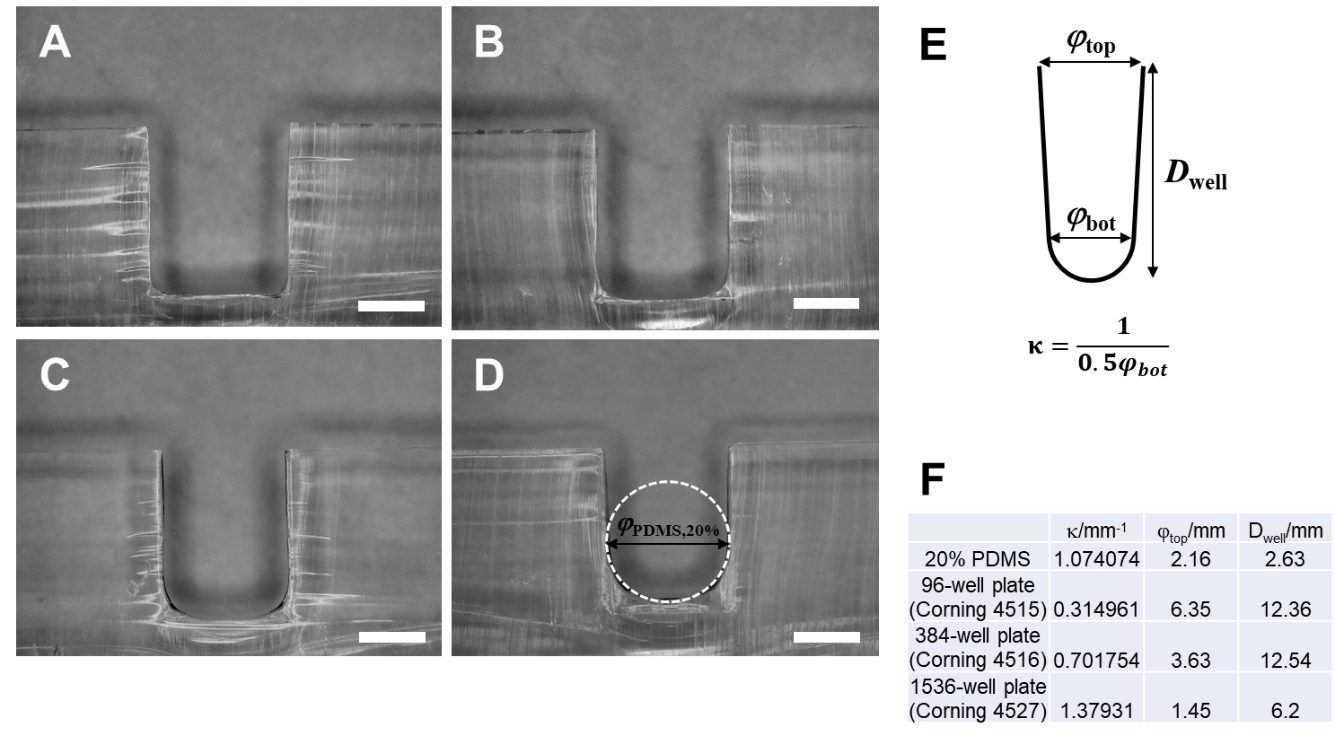
Quantifying curvature (κ) values for PDMS microwells and commercial well plates. Cross-sectional micrographs of PDMS microwells formed with (A) 5% tBA, (B) 10% tBA, (C) 15% tBA, and (D) 20% tBA. (E) Schematic drawing of a well from commercially available microwell plates. (F) Table summarizing curvatures, top diameters and well depths. The curvature of 20% PDMS wells lies between 384- and 1536-well plates. Scale bar represents 1 mm.

3. Supplementary Figure S3


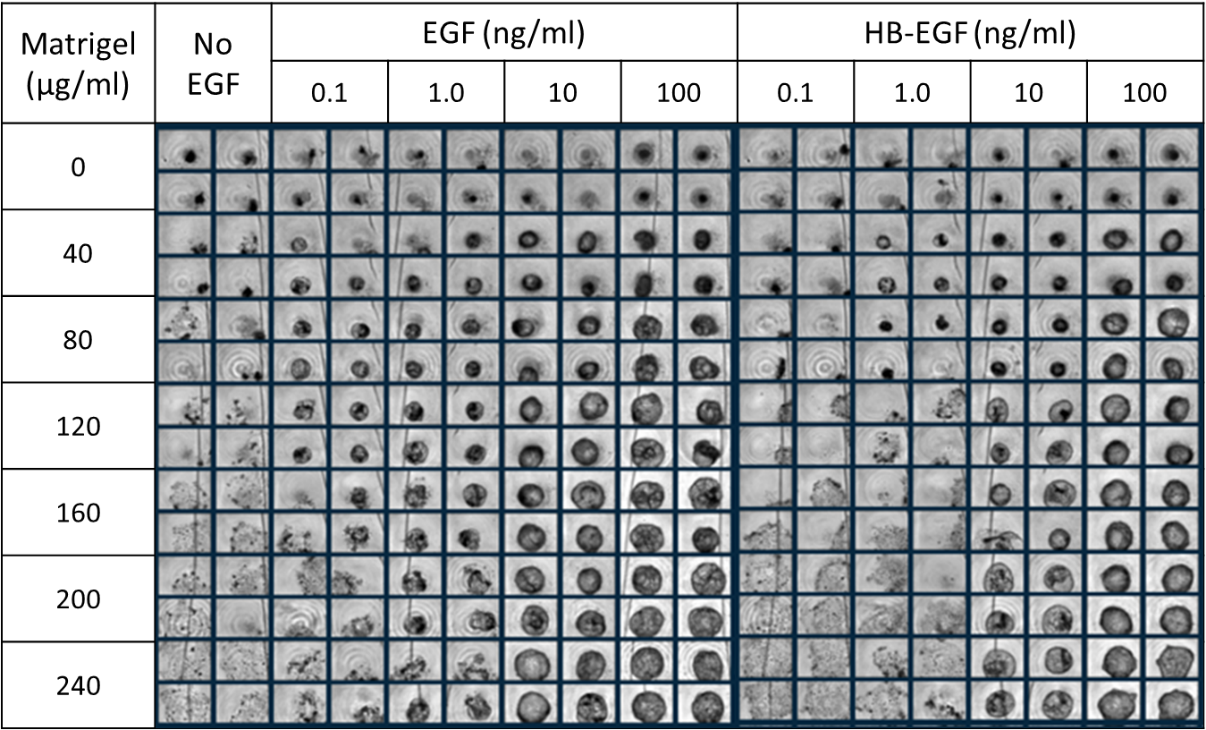
Entire plate images at various concentrations of Matrigel and growth factor on day 16. Organoid formation under various culture conditions with varying amounts of Matrigel and growth factor. The first two columns were maintained without growth factor (EGF or HB-EGF), while the other columns were maintained with EGF or HB-EGF over the full 16 days.

4. Supplementary Figure S4

Scatterplots showing results of Matrigel and growth factor screen. Area and circularity measurements from day 2 (left) and day 16 (right) images are shown. All 63 conditions tested in quadruplicate are depicted in plots.


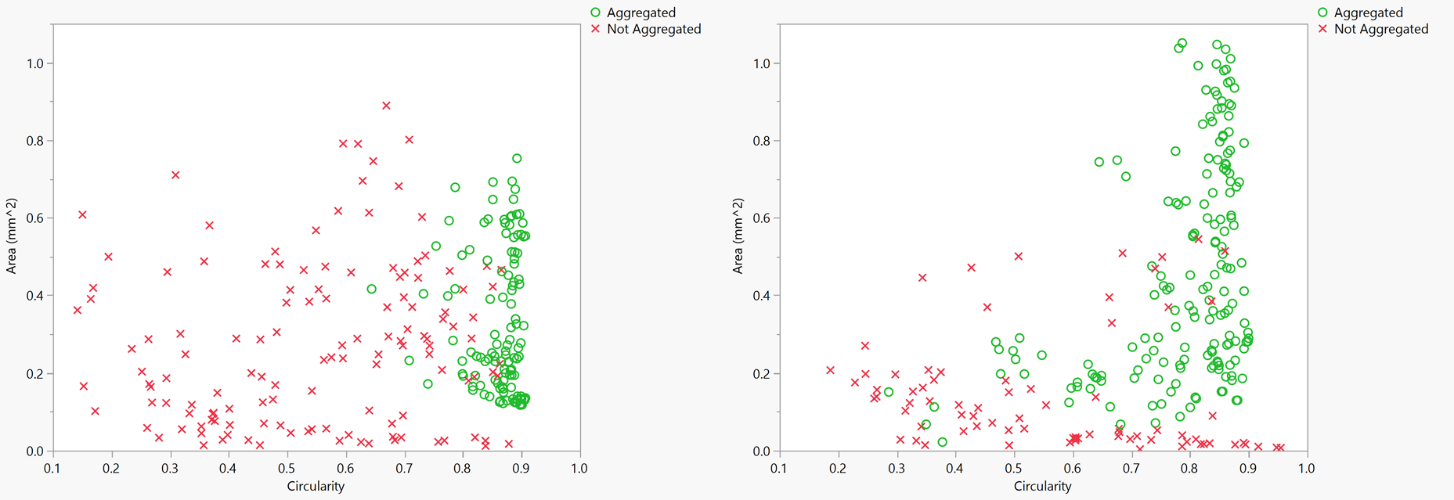


5. Supplementary Figure S5

Partially cell-filled internal structures under 120 µg/mL Matrigel plus 100 ng/mL EGF condition. Laminin-5 and Hoechst 33342 staining of a day 16 organoid section. All scale bars represent 100 μm.


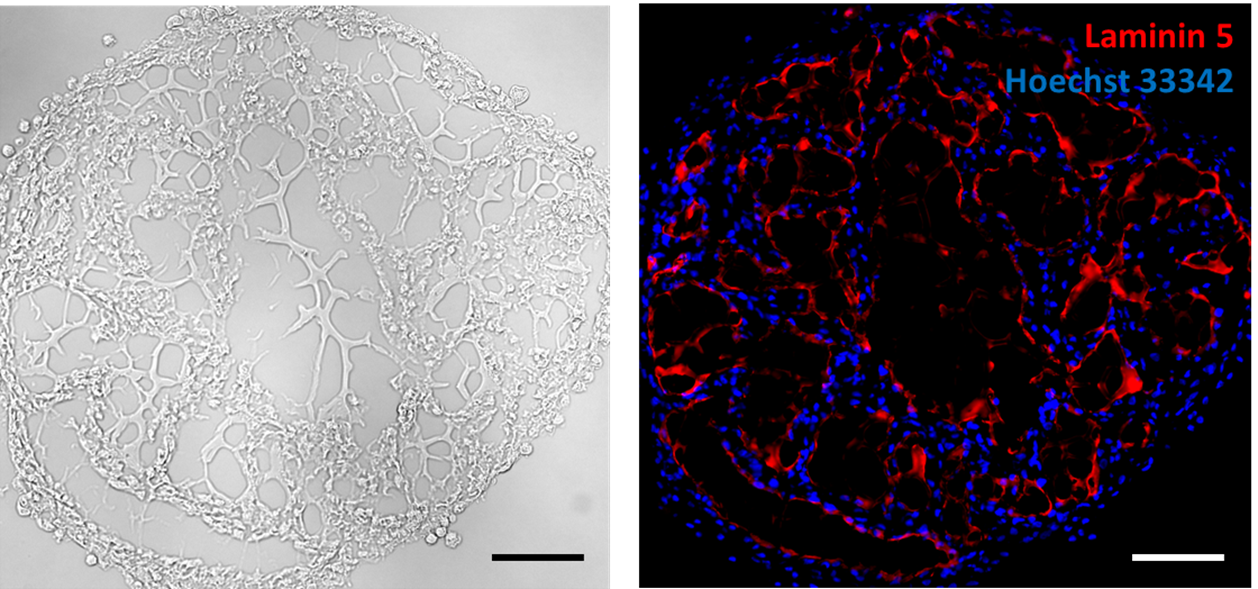


6. Supplementary Video S1

Time-lapse video of organoid formation with or without centrifugation over 24 hours. See video file.


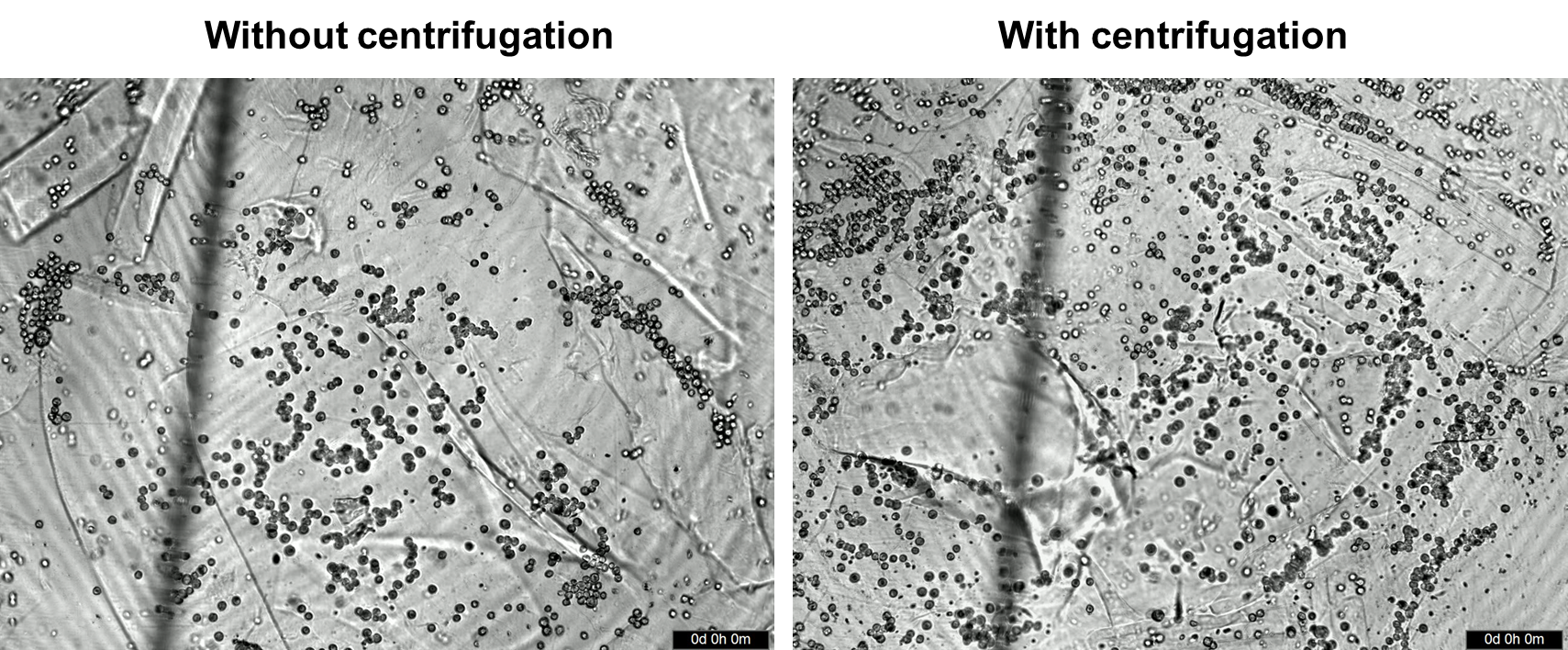


7. Supplementary Method

MATLAB script

%% Organoid Size and Circularity Analysis

% This script will have output variables for organoid area and circularity.

% These variables can be further analyzed in MATLAB or saved for use in

% other data analysis programs

clear all

close all

clc

%% Load in images

% All images should be placed in a folder with this script.

tif_files = dir('*.tif'); % to change to jpg file format, substitute '*.jpg'

filenames = {tif_files.name};

%% Define variables and preallocate for speed

area_output = zeros(1,length(filenames));

circularity_output = zeros(1,length(filenames));

img = imread(filenames{1}); % Read in first image to get size

total_area_pixels = size(img, 1)*size(img, 2); % Area in pixels

total_area_mm2 = 1.74592*1.2896; % this is the total area of a 10X incucyte image

%% Morphological Filtering and Area/Circularity Calculation

for i = 1:1:length(filenames)

% Morphological Image Processing

figure

img = imread(filenames{i});

img = rgb2gray(img); % Convert to grayscale

bw_img = imbinarize(img); % Convert to black and white

bw_img = imcomplement(bw_img); % Invert image

filled_bw_img = imfill(bw_img,'holes'); % Fill holes in mask

SE=strel('disk', 30); % Disk shaped structuring element: radius = 30 (size can be

changed to optimize results)

opened_img = imopen(filled_bw_img, SE); % Morphological opening

closed_img = imclose(opened_img, SE); % Morphological closing

% Show original and processed image

subplot(1,2,1);

imshow(img);

subplot(1,2,2);

[boundaries,L] = bwboundaries(closed_img,'noholes'); % find all discrete objects in

image

imshow(label2rgb(L,@jet,[.5 .5 .5])) % Show processed image

hold on

% Locate largest identified object

if size(boundaries,1)>0

for j=1:1:length(boundaries)

temp(j) = size(boundaries{j},1);

end

[M,largest_object] = max(temp);

else

largest_object=0;

end

temp = 0;

% Find the area and circularity of the largest object (if at least one

% object is detected)

if largest_object > 0

boundary = boundaries{largest_object};

plot(boundary(:,2),boundary(:,1),'w','LineWidth',2) % outline largest object

stats = regionprops(L,'Area','Centroid');

% Identify boundary coordinates

boundary = boundaries{largest_object};

% Calculate object perimeter

delta_sq = diff(boundary).^2;

perimeter = sum(sqrt(sum(delta_sq,2)));

% Calculate area and circularity

area = stats(largest_object).Area;

circularity = 4*pi*area/perimeter^2;

% Results (save these variables)

circularity_output(i) = circularity;

area_output(i) = area/total_area_pixels*total_area_mm2;

else

% If no object is detected, add zeros

circularity_output(i) = 0;

area_output(i) = 0;

end

end
