# Supplementary material for: High-throughput formation and image-based analysis of basal-in mammary organoids in 384-well plates
Source: Sci Rep. 2022 Jan 10;12:317. doi: 10.1038/s41598-021-03739-1 (PMC8748891; doi:10.1038/s41598-021-03739-1)
Supplement: Supplementary file 2 — Supplementary Video S1. [file 41598_2021_3739_MOESM2_ESM.pptx]

## Slide 1
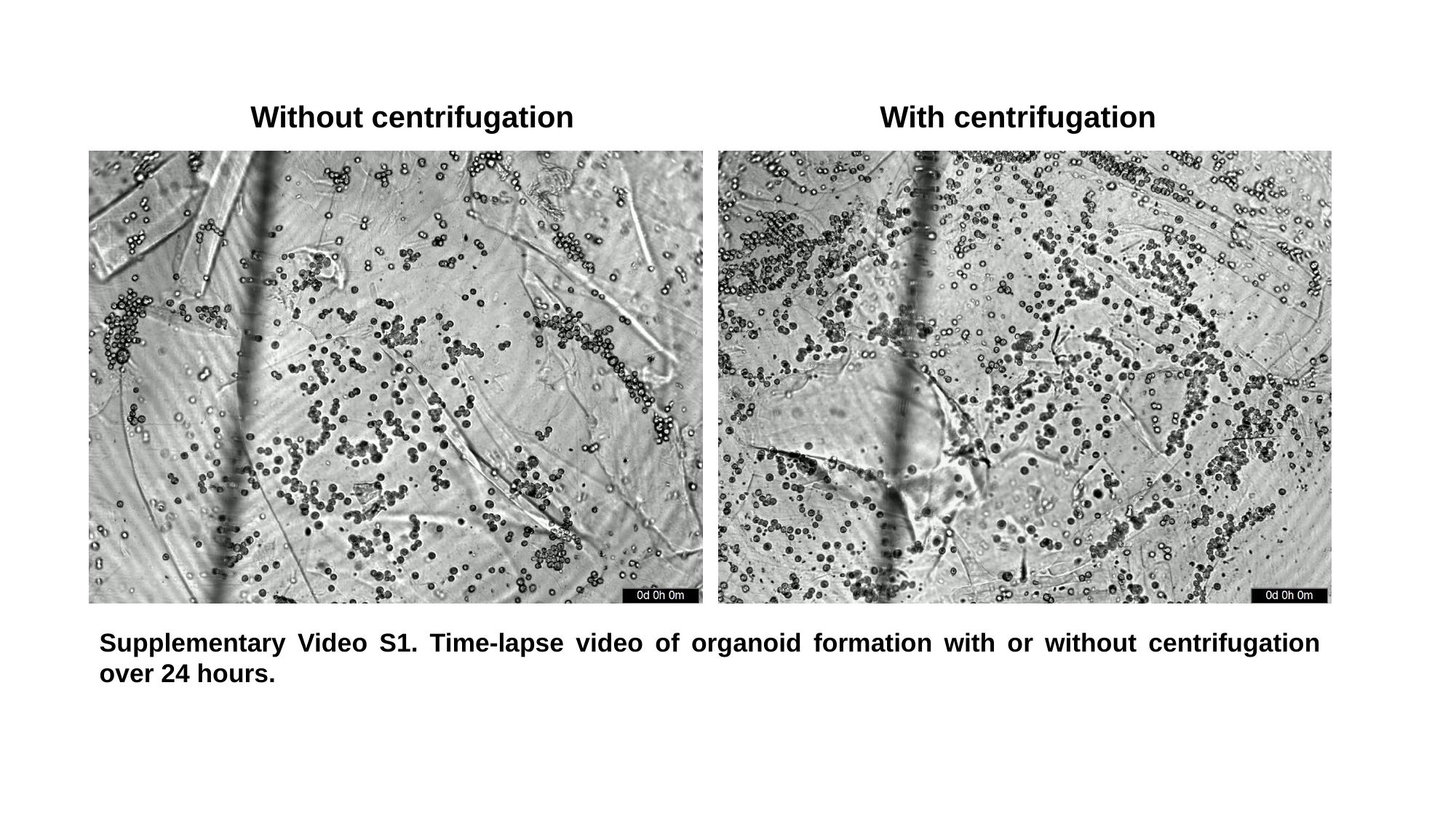

Without centrifugation			 With centrifugation
Supplementary Video S1. Time-lapse video of organoid formation with or without centrifugation over 24 hours.
